# Method validation and risk assessment for sulfonamides and tetracyclines in bees’ honey from Egypt, Libya and Saudi Arabia

**DOI:** 10.1007/s10653-022-01258-0

**Published:** 2022-04-13

**Authors:** Mohamed Bedair M. Ahmed, Amro Ahmed Taha, Fathy Mohamed Saber Mehaya

**Affiliations:** 1 Department of Food Toxicology and Contaminants, National Research Centre, 33 El-Bohouth Street, P. O. box: 12622, Dokki Cairo, Egypt; 2 Beekeeping Research Department, Plant Protection Research Institute, Agricultural Research Center, Dokki Giza, Egypt; 3 Department of Food Technology, National Research Centre, 33 El-Bohouth Street, P. O. box: 12622, Dokki Cairo, Egypt; 4 Research and Training Station, King Faisal University, Al-Ahsa, Saudi Arabia

**Keywords:** Bee’s honey, Contamination, Sulfonamides, Tetracyclines, Chromatographic analysis, Method validation, Risk assessment

## Abstract

Several studies worldwide have reported contamination of bees’ honey by antibiotics, which may pose a hazard to consumers’ health. The present study was thus established to: (1) introduce a validated multi-residue method for determining sulfonamides (SAs) and tetracyclines (TCs) in honey; and (2) characterize the potential risk due to the exposure to SAs and TCs in honey samples from Egypt, Libya, and Saudi Arabia. SAs and TCs were simultaneously extracted using solid-phase extraction and matrix solid phase dispersion methods. SAs and TCs were screened using HPLC–MS/MS and HPLC–DAD. The results confirmed detection limits for SAs and TCs by HPLC–MS/MS of 0.01 and 0.02–0.04 (ng g^−1^), respectively. The limits were 2.5–5.6 and 12.0–21.0 (ng g^−1^) for SAs and TCs by HPLC–DAD, respectively. The obtained accuracy rates were in the ranges of 83.07–86.93% and 86.90–91.19%, respectively, for SAs and TCs, with precision rates lower than 9.54%. Concerning the occurrence of antibiotics, the positive samples constituted 57.6%, 75%, and 77.7% of the Egyptian, Saudi Arabian, and Libyan samples, respectively. Notably, SAs antibiotics were the most prevalent in the Egyptian and Saudi Arabian samples; in contrast, TCs were the most dominant in Libya. Calculated parameters of risk assessment, concerning the aggregated exposure to SAs and TCs, showed no potential adverse effects from the exposure to contaminated honey in studied countries.

**Supplementary Information:**

The online version contains supplementary material available at 10.1007/s10653-022-01258-0.

## Introduction

Honey is a natural sweet substance that is produced from the nectar collected from flowers by honeybees. The honey food matrix is very complicated, in which over 300 chemical substances have been identified (Kujawski & Namieśnik, 2008). It contains multiple vital ingredients, including sugars (glucose and fructose), organic acids, bio-minerals, vitamins, enzymes, hormones and essential oils (Kujawski & Namieśnik, 2008).

Honey is considered an important functional food, showing antimicrobial activity and known to have been used in traditional medicine in ancient Egyptian, Greek, and Roman civilizations (Ransome, 1987). In contemporary studies, it has also been concluded that bees’ honey is useful for managing wounds and gastritis (Kujawski & Namieśnik, 2008).

As bees’ honey is considered a functional food and is expected to be helpful for therapeutic nutrition for many diseases, there is a particular need for it to be devoid of contaminants posing a risk to human health. Numerous contaminants have been detected in bee’s honey such as heavy metals, pesticides, organic pollutants, radioactive isotopes, antibiotics and genetically modified organisms (Baša Česnik et al., 2019; Chiesa et al., 2018; Reybroeck, 2018; Siede et al., 2003). The contamination of honey by antibiotic residues has frequently been reported (Bogdanov, 2006; Galarini et al., 2015; Hammel et al., 2008; Ortelli et al., 2004; Saridaki-Papakonstadinou et al., 2006). This contamination has occurred due to the usage of antibiotics to treat bacterial infections in apiculture, such as American foulbrood (Baran et al., 2011; Oka et al., 2000). Chronic exposure to residual antibiotics in foods of animal origin, such as milk, meat, eggs, and honey, can have adverse effects on public health. Such effects include liver injury, allergic reactions, damage to calcium-rich regions such as bones and teeth, and indirect effects through the development of resistant bacterial strains (Johnson et al., 2010; Pataro et al., 2003). These resistant bacteria might then impede human treatments. The WHO has classified antibiotic resistance as “one of the greatest threats to public health” (WHO, 1997).

Sulfonamides (SAs) and tetracyclines (TCs) are the most important antibiotics used in commercial apiculture, with broad-spectrum effects against bacteria (Johnson et al., 2010; Mutinelli, 2003; Oka et al., 2000). The improper use of SAs and TCs can lead to contamination of honey samples with such antibiotics as reported by the international studies (Baggio et al., 2009; Chen et al., 2001; Hammel et al., 2008; Johnson et al., 2010; Mahmoudi et al., 2014; Orina, 2014; Saleh et al., 2016; Saridaki-Papakonstadinou et al., 2006; Tantillo et al., 2000).

At present, there are no assigned maximum residue limits (MRLs) for antibiotics in honey in Egypt, Libya and Saudi Arabia. However, some countries, such as Belgium, Switzerland, and the UK, have established action limits for antibiotics in honey, which generally lie between 10 and 50 µg/kg for each antibiotic group (Bogdanov, 2006). In particular, the Swiss authorities have set MRLs for SAs and TCs in honey of 50 and 20 µg/kg, respectively (Dluhošová et al., 2018; Koesukwiwat et al., 2007b).

Multi-residue methods for the analysis of veterinary drugs in foods of animal origin are highly recommended for periodical screening programs, in which time-saving is particularly important. Sample pretreatments are essential procedures for residue analysis. Purification methods for determining SAs and TCs residues in honey samples often include several steps, such as liquid–liquid extraction (LLE) and solid-phase extraction (SPE) (Verzegnassi et al., 2002; Zotou & Vasiliadou, 2006). The matrix solid-phase dispersion (MSPD) process combines extraction and purification procedures (Kristenson et al., 2006), which has been proven to be a good alternative to liquid–liquid extraction (LLE) for solid or semi-solid samples (Kristenson et al., 2006; Zhang et al., 2005).

Several authors tried to development selective and accurate method to pretreat the honey samples in order to avoid the matrix interference. In this concern, Koesukwiwat et al. (2007a) extracted SAs and TCs from bovine milk using McIlvain’s buffer (pH 4.5), followed by a clean-up step by SPE using an Oasis HLB 200-mg cartridge. Bohm et al. (2012) extracted TCs from honey using EDTA MachIlvien’s buffer (pH 4), followed by clean-up on an OASIS HLB SPE cartridge (200-mg). Zou et al. (2008) used C_18_ as a MSPD sorbent for the extraction of SAs from honey. Meanwhile, Zhang et al. (2012) used diatomaceous earth to extract SAs from blood samples following the MSPD technique.

To our knowledge, this is the first study to estimate and evaluate the residual contents of SAs and TCs in honey samples from Egypt, Libya, and Saudi Arabia. The present investigation aimed: (1) to introduce a multi-residue method for determining SAs and TCs in bees’ honey using either HPLC-diode array detector (DAD) or HPLC–MS; (2) quality assurance of the proposed methods of analysis; (3) to study the occurrence of SAs and TCs residues in bees’ honey from three Arabian countries (Egypt, Libya and Saudi Arabia); and (4) to assess the potential risk due to the exposure to SAs and TCs in the studied samples.

## Materials and methods

Different samples of honey were collected randomly from three Arabian countries with different environments in different seasons.

### Sampling

Eighty-four samples of bees’ honey were collected from within 3 countries, Egypt, Libya, and Saudi Arabia, as follow: from 7 regions in Egypt (33 samples, representing 39.3% of the total samples), 8 regions in Libya (33 samples, 39.3%), and 6 regions in Saudi Arabia (18 samples, 21.4%). Figure S1 shows the geographic locations of sampling in the three countries. The collected honey samples were pure, unfiltered and unprocessed based on the obtained information from the beekeepers or honey suppliers to supermarkets in each of the studied regions. The collected samples were of different categories belonging to the following diverse floral origins: clover, citrus, thyme, sider, harmal, talh and summra (Table S1). The collected samples were directly extracted and stored in 250 mL fine plastic containers duly labeled with identification code, name and date of collection. The samples were then stored in a dry dark place at 20 °C until further analysis to avoid the laboratory conditions having an effect on the chemical composition and physical properties of the honey samples (Abdalla, 2015; EU-Council, 2002; Godshall, 2003).

### Chemicals and reagents

Diatomaceous earth or Celite (approximately 400 meshe) was obtained from Sigma-Aldrich Corporation (Saint Louis, MO, USA). SPE cartridges, 6 mL/200 mg of HLB (hydrophilic–lipophilic–balanced), were purchased from Waters Oasis Co., (Milford, MA, USA). In addition, 0.45 µm nylon filters, 13 mm in diameter (Agilent Technologies, Palo Alto, CA, USA), were obtained.

Standards of sulfamethazine (SMT), sulfamethoxazole (SMX), sulfadimethoxine (SDM), tetracycline hydrochloride (TC), oxytetracycline hydrochloride (OTC), and chlortetracycline hydrochloride (CTC) were obtained from Sigma-Aldrich.

Stock solutions of antibiotics (100 mg L^−1^) were prepared as follows: 10 mg of individual standard was accurately weighed, dissolved in a small amount of methanol, diluted to 100 mL with methanol (HPLC-grade), and then stored at − 20 °C. From these stock solutions, working solutions were freshly prepared by gradient dilution with methanol (HPLC-grade).

McIlvain’s buffer solution was prepared by dissolving 11.8 g of citric acid monohydrate, 13.72 g of Na_2_HPO_4_, and 33.62 g of Na_2_EDTA in of distilled water. The buffer pH was adjusted to 4.50 and the volume completed to 1 L. This solution was prepared daily and used freshly.

All chemicals and solvents used in the study were of analytical grade or HPLC-grade.

### Extraction procedures

#### *Extract* 1

The MSPD technique reported by Zhang et al. (2012) was used to extract SAs from honey samples with slight modifications. An aliquot 3 g of honey sample was placed in an agate mortar and 3 g of diatomaceous earth was added to the honey sample. The mixture was blended until complete dispersion using a pestle. The homogenized mixture was afterward transferred into a plastic syringe column (10 × 1.5 cm i.d.) with a compacted thin layer of cotton in the bottom of the column. A thin layer of compacted cotton was also added at the top of the sample mixture. The MSPD column was eluted using 10 mL acetone followed by 10 mL ACN (two eluting solvents were applied instead of only one (acetone or ACN was used as an individual trial in the original method). The elusion solvent mixture (acetone and ACN) was evaporated to dryness at 38 °C under vacuum. The dried residue was re-dissolved in 1 mL of HPLC grade methanol and the solution was filtered through 0.45 µm filter membrane and then subject to HPLC analysis.

#### *Extract* 2

TCs in honey samples were extracted following the methodology of Koesukwiwat, et al. (2007b) with slight modifications. First, 5.0 mL of 10% TCA (instead of 2 mL of 20% TCA) was added to 5 g of honey in a 50 mL Falcon tube and vortexed for 1 min. Thirty milliliters (instead of 20 mL) of McIlvain’s buffer (pH 4.5) was added to the mixture, vortexed for 1 min at high speed, and then sonicated for 10 min at high power (a new procedure, instead of manual shaking for 5 min) using an ultrasonic (J.P. SELECTA, Spain). The mixture was centrifuged at 4500 rpm for about 20 min and the supernatant was subjected to SPE clean-up.

The supernatant of the honey extract from the above procedures was passed through an Oasis HLB 200 mg cartridge (preconditioned with 5.0 mL of methanol, 5.0 mL of 0.5 N HCl, and 5.0 mL of de-ionized water) for the SPE clean-up. The cartridge was sequentially washed with 5.0 mL of methanol (5%) (CH_3_OH: H_2_O, 5:95%) followed by 5.0 mL of methanol (5%) containing 2% acetic acid. Subsequently, the cartridge was air dried under suction for 5 min. The analyte was sequentially eluted from the cartridge into a 30 mL rotary round—bottomed flask using 5.0 mL of methanol (absolute), followed by 5.0 mL of methanol (5%) containing 2% NH_4_OH. The extract was evaporated, under vacuum at 38 °C to dryness using a rotary evaporation system. The dried residue was re-suspended in 1.00 mL of methanol (HPLC grade) followed by filtration through a 0.22 µm filter before being subjected to HPLC analysis. Twenty microliters (10 µL of each of extract 1 and extract 2) were injected into the HPLC system for analysis.

### Screening procedures

#### HPLC–DAD

The HPLC–DAD system used for the determination of SAs and TCs was an Agilent 1100 series for high-performance liquid chromatography (Agilent Technologies, Waldbronn, Germany). The mobile phase gradient program is applied as shown in Table S2. The system consisted of a vacuum solvent degassing unit, a quaternary gradient pump, an automatic sample injector, and DAD. The LC separation column was an Eclipse XDB-C18 (150 × 4.6 mm I.D., 5 µm particle size), Eclipse; USA. A Phenomenex (Torrance, USA) guard column (3.0 × 4 mm) of the same material was also used. Mobile phase A was composed of ACN (HPLC grade) containing 0.01 M oxalic acid and 0.1% formic acid, and mobile phase B was 0.03 M oxalic acid, pH 2.3. The flow rate and sample run time were 1.0 mL min^−1^ and 32 min, respectively. The injection volume was 20 µL. DAD was adjusted to measure SAs and TCs at wavelengths of 280 and 365 nm, respectively.

### HPLC–MS/MS

The HPLC–MS/MS system used was an Agilent 1260 series for high-performance liquid chromatography-tandem mass spectrometry (Agilent Technologies, Palo Alto, CA, USA) equipped with a variable wavelength UV detector. Positive electrospray ionization (ESI) mode was used to detect SA and TC antibiotics. The SA and TC determination was conducted following the method described by Ahmed et al. (2015). Specifically, the LC column used was YMC-Pack Pro C18 RS (150 × 2.0 mm I.D., 3 µm particle size). The used mobile phase A consisted of HPLC grade water and formic acid (99.9:0.1 v v^−1^, pH 2.6), while mobile phase B was HPLC grade acetonitrile and formic acid (99.9:0.1 v v^−1^). The gradient program for separation is shown in Table S1. The column was maintained at 25 °C with a flow rate of 0.2 mL min^−1^. The injection volume was 20 µL.

Conditions of the mass spectrometry (MS) analysis: The used MS detector was a Thermo Finnigan LCQ Duo ion trap mass spectrometer (Thermo, Woburn, MA, USA), equipped with a heated capillary interface and ESI. Ultra-pure nitrogen gas was used for drying and nebulizing. Spray voltage was set to 4.5 kV and the capillary voltage was maintained at 3500 V. Drying gas temperature was set at 350 °C, and the flow rate was 10.0 L min^−1^.

### Method quality assurance

#### Linearity

The linearity of response to antibiotic pure standards was examined for HPLC–DAD and HPLC–MS/MS using concentration ranges of 50–1000 ng and 1–100 ng, respectively with five concentrations for each antibiotic. Each concentration was prepared in triplicate for each antibiotic. Calibration curves for SAs and TCs were created by plotting the peak area against the antibiotic concentration (ng) using Microsoft Excel 2010. Linearity equations and *r*^2^ were obtained.

#### Detection limit (DL) and quantification limit (QL)

The DL and QL were determined using equations of the calibration method (Guideline, 2005; Obakpororo et al., 2017).

$${\text{DL}} = \frac{3.3 \times \sigma }{S}\;\;\;\;\;{\text{QL}} = \frac{10 \times \sigma }{S}$$

Here, *σ* = the standard deviation of the response or standard deviation of *y*-intercepts. *S* = the slope of the calibration curve.

#### Accuracy and precision

The accuracy and precision values were estimated to evaluate the extraction method. The accuracy values were studied in triplicate analyses using honey samples (free of antibiotics). Honey samples were spiked with the six antibiotics at a concentration of 50 µg kg^−1^, which equals the MRLs set by the Swiss authorities. The spiked samples were left for 1 h in the dark before extraction. The accuracy rate was calculated according to the following equation:

$$ {\text{ Accuracy ratio (\% ) = }}\left( {\frac{{\text{Measured content}}}{{\text{Fortification level}}}} \right) \, \times 100 $$

Precision (repeatability) was estimated by calculating the relative standard deviation rate (RSD %) for each antibiotic accuracy using three replicates.

$$ {\text{ RSD (\% ) = }}\left( {\frac{{{\text{SD}}}}{{\text{Average value}}}} \right) \, \times 100 $$

### Risk assessment for human exposure to antibiotics in honey

#### Exposure assessment

Exposure assessment of SAs and TCs in honey was based on the mean detected concentrations in samples. The estimated daily intake (EDI) of SAs and TCs as µg/day was calculated using the following equation:

$${\text{EDI}} = \frac{{{\text{Cf}} \times {\text{Occ}}}}{{{\text{BW}}}}$$

Here: ${\text{Cf}}$ is the daily honey consumption (g/person/day), ${\text{Occ}}$ is the occurrence of antibiotic in honey (average concentration as ng/g honey), and ${\text{BW}}$ is the mean body weight of an adult (70 kg). The average of annual consumption of bees’ honey per capita in Egypt is 1 kg (Al Naggar et al., 2018). By dividing 1 kg honey across 365 days, the daily consumption per capita is 2.7 g day^−1^. Calculated in the same way, the daily consumption of honey in Libya is 9.8 g day^−1^ per capita (Faostat, 2016). While in Saudi Arabia, it is 12.3 g day^−1^ (Zulail et al., 2014).

Adult Egyptians’ daily consumption rates of other food items in which SAs and TCs might be found are as follows: cattle meat (29.89 g), poultry meat (29.31 g), eggs (19.8 g), fresh milk (180 g), and fish (97.88 g) (CAPMAS, 2021; Wally, 2016). The corresponding values in Libya and Saudi Arabia are as follows: cattle meat (33 and 139 g), poultry meat (43 and 103 g), fish (19 and 23.25 g), fresh milk (111 and 241 g), and eggs (24.6 and 12.25 g), respectively (Adam, et al., 2014; Ahmed, et al., 2019; Moradi-Lakeh, et al., 2017; Ritchie & Roser, 2017; Selvanathan, et al., 2016).

#### Risk characterization

Representative parameters to characterize the potential risk of a certain compound are the MRLs and the acceptable daily intake (ADI). We have used the assigned MRLs by the Swiss authorities, which are 50 and 20 µg/kg for SAs and TCs, respectively (Dluhošová, et al., 2018; Koesukwiwat, et al., 2007b). The ADIs for SAs and TCs were 3 and 5 (µg kg^−1^ bw day^−1^), respectively, according to the FAO/WHO (2010).

The new approaches for risk characterization were performed following the equations presented by Goumenou and Tsatsakis (2019) for single chemicals and for chemical mixtures. The new targeted parameters are the source-related hazard quotient (HQ_S_) and hazard index (HI_S_), as well as the adversity-specific hazard index (HI_A_).

HQ_S_ was calculated according to the following equation:

$${\text{HQs}} = \frac{{\text{EXP aggregated}}}{{{\text{ADI}}}}$$

$${\text{EXP aggregated = }}\frac{{\text{EXP froms pecific food item}}}{{\text{Correction factor (CF)}}}$$

$${\text{CF }} = \, \frac{{\left( {{\text{Consumption of specific food }} \times {\text{ MRL in the specific food}}} \right)}}{{\sum\nolimits_{i}^{n} {\left( {{\text{Consumption of food }}i \, \times {\text{ MRL in the food }}i} \right)} }}$$

Here: *n* refers to the number of food items in which SAs and TCs might be found (Five food items previously mentioned above in the exposure assessment section). The equation numerator represents the consumed amount of honey multiplied by the MRL, while the denominator refers to the sum of the calculated values of the other five food items representing the whole diet. The calculated *CF* value for SAs antibiotics was 0.0037, while it was 0.0014 for TCs.

Regarding the risk characterization for a mixture of *n* chemicals (3 SAs and 3 TCs) in a specific food item (honey), the source related HI_S_ was calculated based on the following equation:

$${\text{HIs = }}\sum\limits_{i}^{n} {\left( {{\text{HQs}}} \right)} \, i$$

Here, *n* represents the number of chemicals in the mixture.

HI_A_ was calculated according to the following equation:

$${\text{HIA}} = \sum  \limits_{i}^{\text{n}} {\text{HQ i}}  = \sum \limits_{i}^{\text{n}} \left( {\frac{\text{EXP i}}{\text{ADI i}}} \right)$$

Here, we considered the sum of the three SAs and the sum of 3 TCs because antibiotics from the same family have the same mode of action.

## Results and discussion

### Quality assurance of HPLC–DAD and HPLC–MS/MS

The data presented in Table 1 shows the sensitivity and linearity parameters for HPLC–DAD and HPLC–MS/MS when determining SAs and TCs. Linear correlations were found between the analyte’s concentration and the peak area of response by HPLC instruments. *R*^2^ values, for both SAs and TCs, were between 0.978 and 1.00. These results confirmed the validation of both instruments in the analysis of SAs and TCs. These results are in agreement with our previous studies (Ahmed et al., 2015, 2020).

**Table 1:** Linearity range and sensitivity of instruments

Antibiotics		*T*_R_ (Min) ± SD (*n* = 10)	Linear equation	(*R*^2^)	DL (ng g^−1^)	QL (ng g^−1^)
*HPLC–DAD system*						
SAs	SMT	05.72 ± 0.10	*y* = 4.614*x* − 16.41	0.999	05.6	15.0
	SMX	15.12 ± 0.50	*y* = 3.510*x* − 5.630	1.000	03.5	06.5
	SDM	22.25 ± 1.04	*y* = 3.701*x* − 4.131	0.999	02.5	06.0
TCs	OTC	09.02 ± 0.33	*y* = 0.502*x* − 2.041	0.999	12.0	21.5
	TC	10.42 ± 0.38	*y* = 0.612*x* − 2.595	0.999	13.0	24.0
	CTC	16.28 ± 0.64	*y* = 0.402*x* − 2.697	0.995	21.0	30.0
*HPLC–MS/MS system*						
SAs	SMT	19.56 ± 0.09	*y* = 4000000*x* + 41,433	0.999	0.01	0.03
	SMX	30.37 ± 0.15	*y* = 3000000*x* + 71,372	0.978	0.01	0.02
	SDM	36.58 ± 0.13	*y* = 5000000*x* + 200,000	0.998	0.01	0.02
TCs	OTC	16.11 ± 0.13	*y* = 61536*x* − 37,579	0.999	0.03	0.09
	TC	17.32 ± 0.16	*y* = 76419*x* − 100,000	0.985	0.02	0.06
	CTC	24.62 ± 0.50	*y* = 40078*x* − 44,682	0.989	0.04	0.12

Significant differences in the values of DLs and QLs were markedly observed between the DAD and MS/MS detectors. The DLs for MS/MS ranged between 0.01 ng g^−1^ for SAs and 0.02–0.04 (ng g^−1^) for TCs which were lower than the DLs for DAD by 250 (SDM)–650 folds (TC). The QLs of MS/MS for SAs and TCs were also markedly lower by values ranged between 239 (OTC) and 500 (SMT) times less than those of DAD.

The QLs of DAD for SAs and TCs (0.6–15 ng g^−1^ and 21.5–30 ng g^−1^, respectively) were also notably lower than the permissible limits assigned by the Swiss authorities (50 and 20 ng/g, respectively). Therefore, HPLC–DAD, in the proposed method of current study, could recover the trace concentrations of antibiotic-contaminated honey and can be used for the periodical inspection programs for SAs and TCs in honey.

**Fig. 1:**
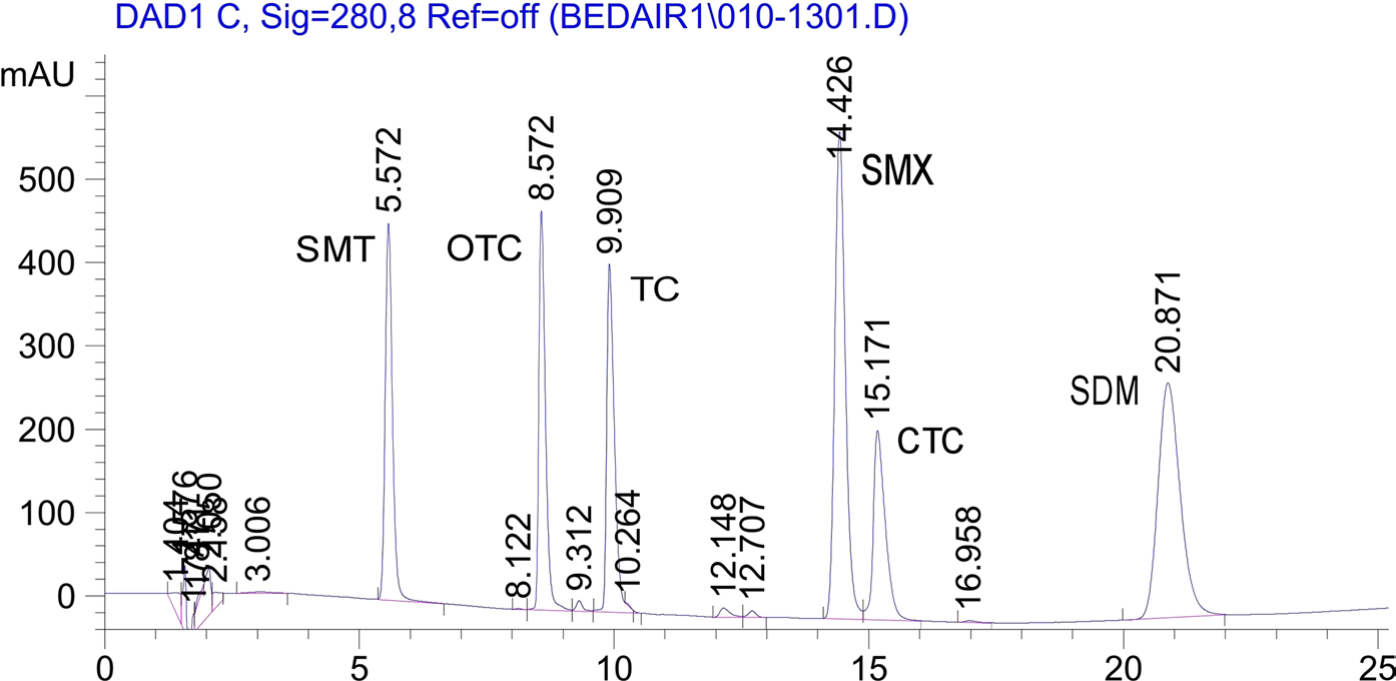
Typical HPLC–DAD chromatogram for the separated SAs and TCs

**Fig. 2:**
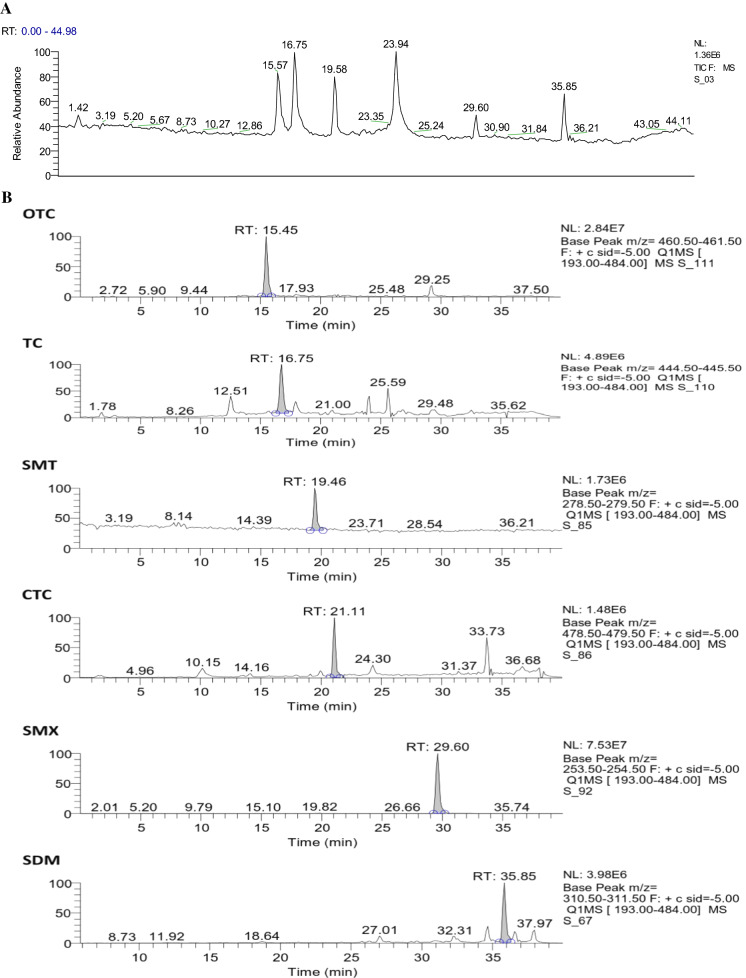
HPLC–MS/MS: **a** TIC for SAs and TCs; **b** m/z for every antibiotic

The proposed chromatographic methods of separation (Table S2) proved their selectivity for SAs and TCs compounds and the easier distinction between them and other potential interfering compounds. Data in Table 1 and the chromatograms shown in Figs. 1, 2 and S2 indicate the *T*_R_ values of separated antibiotics, which confirmed that the adopted method is valid to avoid any interfering molecules under the same peak.

### Quality assurance of the extraction methods

Owing to chemical differences between SAs and TCs, significant differences in accuracy rates were found following the two methods of extraction (Table 2). When using EDTA MachIlvien’s buffer, SAs showed lower accuracy values (57.81–65.63%) than TCs (86.90–91.19%). These results agreed with the findings of Bohm et al. (2012), who reported high accuracies for TCs (93–95%), when extracted from honey with EDTA MachIlvien’s buffer (pH 4), followed by clean-up using an Oasis HLB 200-mg cartridge. Acidified EDTA MachIlvien’s buffer was more appropriate for the extraction of TCs. Since TCs have a complicated structure containing many binding sites in their electron-rich groups, such as carbonyl, dimethylamine and hydroxyl groups, TCs can form complexes with different metal ions in honey matrix which makes the extraction of TCs difficult (Guerra et al., 2016). However, when using EDTA MachIlvien’s buffer, the EDTA salt strongly chelates the metal ions (Mohammadi et al., 2013) in honey while the buffer solution easily extracts the TCs (Anderson et al., 2005).

**Table 2:** Accuracy and precision rates of the extraction methods

Antibiotics		EDTA MachIlvien’s buffer method		MSDP method	
		Accuracy (%) ± SD	RSD (%)	Accuracy (%) ± SD	RSD (%)
SAs	SMT	65.63 ± 5.56	8.47	84.65 ± 8.08	9.54
	SMX	64.79 ± 6.14	9.48	86.93 ± 5.84	6.72
	SDM	57.81 ± 5.22	9.03	83.07 ± 6.80	8.19
TCs	OTC	91.19 ± 4.25	4.67	50.20 ± 2.18	4.33
	TC	86.90 ± 5.55	6.39	45.48 ± 4.07	8.96
	CTC	90.43 ± 4.98	5.51	46.83 ± 3.80	8.12

^*^RSD relative standard deviation

In contrast, the extraction of honey samples using MSDP by diatomaceous earth proved highly capable of extracting SAs with accuracy rates of 84.65%, 86.93%, and 83.07% for SMT, SMX, and SDM, respectively. These results are in agreement with the work of Zou et al. (2008) who reported accuracy of over 70% for SAs, when extracted from honey using C_18_ as a MSPD. Meanwhile, MSDP with diatomaceous earth exhibited lower recovery rates with TCs (45.48–50.20%). Because diatomaceous earth mainly consists of 80–90% silica, 2–4% alumina, and 0.5–2% iron oxide, Al and Fe ions may negatively affect the extraction of TCs, resulting in low accuracy.

Notably, the precision and repeatability values of the two applied methods were quite accepted, RSD rates were markedly lower than the recommended value (15%) assigned by the EU directive (2002/657/EC) concerning the performance of analytical methods.

Based on the obtained data in Table 2, extracts of both EDTA buffer and MSDP methods were combined to ensure high accuracy and precision values, and such justification was applied in the occurrence study of SAs and TCs in honey samples of Egypt, Libya and Saudi Arabia.

### Occurrence of SAs and TCs in Egyptian honey

Data in Table 3 revealed that samples from both Beheira and Ismailia regions were positive for the occurrence of SAs (SMT and SMX) with concentrations below 52 µg kg^−1^. SMT and SMX showed high incidence in all Egyptian samples (100%), while SDM was not detected anywhere. The examined samples from the regions of Dakahlia, Gharbia, Menofia and Qalyubia contained multiple antibiotics from both SAs and TCs groups. The highest averages of the detected concentrations of SMT and SMX were scored for Gharbia samples and Giza samples, respectively as 23.5 and 86.9 µg kg^−1^. This means that SMX was the most used antibiotic from the SAs group. On the other hand, the highest averages of the detected TCs were scored by samples from Dakahlia (OTC: 38.72 µg kg^−1^ and TC: 131.75 µg kg^−1^) and Qalybia (CTC: 58.18 µg kg^−1^). This revealed that TC could be the most used member of the TCs group followed by CTC then OTC. In terms of frequency, of positivity, the antibiotics were found in the following order: SMT = SMX > TC > CTC > OTC > SDM.

**Table 3:** Occurrence of SAs and TCs in Egyptian honey

Region	Total/positive samples (frequency %)	Conc	Concentrations (µg kg^−1^)					
			SMT	SMX	SDM	OTC	TC	CTC
Beheira	**4/1** (25)	Average	**0.308**	**3.847**	< d.l. *	** < d.l**	** < d.l**	** < d.l**
		Max	0.308	3.847	–	–	–	–
Dakahlia	**6/3** (50)	Average	**1.463**	**3.077**	** < d.l**	**38.722**	**131.754**	**29.759**
		Max	3.997	8.887	–	39.849	146.002	–
Gharbia	**4/3** (75)	Average	**23.514**	**11.280**	** < d.l**	**30.896**	**94.392**	** < d.l**
		Max	70.052	33.176	–	30.896	94.392	–
Giza	**4/4** (100)	Average	**8.974**	**86.895**	** < d.l**	** < d.l**	**85.426**	**33.778**
		Max	28.817	275.080	–	–	85.426	46.055
Ismailia	**5/1** (20)	Average	**1.037**	**51.550**	** < d.l**	** < d.l**	** < d.l**	** < d.l**
		Max	1.037	51.550	–	–	–	–
Menofia	**4/3** (75)	Average	**0.208**	**2.609**	** < d.l**	** < d.l**	** < d.l**	**38.869**
		Max	0.231	–	–	–	–	42.419
Qalyubia	**6/4** (66.7)	Average	**0.832**	**13.519**	** < d.l**	** < d.l**	**75.729**	**58.184**
		Max	2.308	32.445	–	–	77.329	–
Total	**33/19** (57.6)	Main average	**5.191**	**24.682**	** < d.l**	**34.809**	**96.825**	**40.147**
Swiss MRL		(µg kg^−1^)	**50**	**50**	**50**	**20**	**20**	**20**

* < d.l.: below the detection limit

Notably, 57.6% of samples (19 out of 33) were positive for antibiotics. In terms of the frequency of positive samples, the tested regions can be arranged in the following order: Giza > Gharbia = Menofia > Qalyubia > Dakahlia > Ismailia.

To the best of our knowledge, only one Egyptian study, by Abd Alla (2020), determined TC beside chloramphenicol and tylosin in Egyptian honey samples. This study reported lower concentrations of TC ranged between 1.2 ± 0.6 and 6.6 ± 0.4 (µg kg^−1^).

### Occurrence of SAs and TCs in Libyan honey

The overall use of antibiotics in Libyan apiculture can be deduced from the data presented in (Table 4). The results reveal that Libyan beekeepers basically use TC antibiotics to treat bacterial diseases in bees. Apart from very low concentrations of SMT detected only in Gheryan and Wadi Al Hayaa, all samples were free of SAs. In contrast, TCs were widely used antibiotics and all samples contained elevated concentrations of them of over 20 µg kg^−1^ (Swiss MRL). The detected concentration of OTC did not exceed 40 µg kg^−1^, while the elevated average of TC and CTC levels were within the ranges of 74.83–239.36 µg kg^−1^ and 50.42–322.24 µg kg^−1^, respectively. The highest detected concentrations of TC and CTC were recorded for the samples from Tarhounah (269.35 µg kg^−1^) and Khoms (462.48 µg kg^−1^), respectively. The results showed that the frequencies of TC congeners were in the following order: CTC > TC > OTC.

**Table 4:** Occurrence of SAs and TCs in Libyan honey

Region	Total/positive samples (frequency %)	Conc	Concentrations (µg kg^−1^)					
			SMT	SMX	SDM	OTC	TC	CTC
Al Aziziyah	**3/1** (33.3)	Average	< d.l. *	** < d.l**	** < d.l**	** < d.l**	**152.811**	**167.601**
		Max	–	–	–	–	–	–
Bani Walid	**3/2** (66.6)	Average	**0.054**	** < d.l**	** < d.l**	** < d.l**	**107.347**	** < d.l**
		Max	–	–	–	–	185.161	–
Gheryan	**3/3** (100)	Average	** < d.l**	** < d.l**	** < d.l**	**25.363**	**127.919**	**127.090**
		Max	–	–	–	–	168. 161	198.854
Khoms	**3/3** (100)	Average	** < d.l**	** < d.l**	** < d.l**	**21.337**	**150.177**	**322.243**
		Max	–	–	–	–	218.354	462.476
Msallata	**3/3** (100)	Average	** < d.l**	** < d.l**	** < d.l**	**31.661**	**108.013**	**146.402**
		Max	–	–	–	38.651	167.601	240.351
Tarhounah	**3/3** (100)	Average	** < d.l**	** < d.l**	** < d.l**	** < d.l**	**239.365**	**140.453**
		Max	–	–	–	–	269.357	189.565
Tripoli	**3/2** (66.6)	Average	** < d.l**	** < d.l**	** < d.l**	** < d.l**	**74.831**	**50.426**
		Max	–	–	–	–	79.836	–
Wadi al Hayaa	**3/1** (33.3)	Average	**0.081**	** < d.l**	** < d.l**	** < d.l**	** < d.l**	** < d.l**
		Max	–	–	–	–	–	–
Total	**24/18** (75)	Main average	**0.067**	** < d.l**	** < d.l**	**26.120**	**137.209**	**157.323**
Swiss MRL		(µg kg^−1^)	**50**	**50**	**50**	**20**	**20**	**20**

* < d.l.: below the detection limit

Notably, 75% of samples (18 out of 24) were positive for antibiotics and 87.5% of the positive samples had concentrations over the Swiss permissible limits. Moreover, 66.6% of samples contained multiple TCs congeners, which means that Libyan beekeepers used TCs as a mixtures or cocktails. Briefly, the tested regions in Libya can be arranged, in terms of the frequency of positive samples, as follows: Tarhounah = Khoms = Gheryan = Msallata > Bani Walid > Tripoli > Al Aziziyah > Wadi al Hayaa.

### Occurrence of SAs and TCs in Saudi Arabian honey

The results for Saudi Arabian samples differed from those from Libya. Specifically, in Saudi Arabia, SMT and SMX (SAs group) were the most abundant antibiotics found at a rate of 78.5% (11 out of 14 positive samples). TC and CTC were the third and fourth most common after SMT and SMX. Notably, all of the detected levels of SAs were markedly lower than the Swiss legislated limit (50 µg kg^−1^). On the other hand, for TCs, TC exhibited the highest detected concentrations of over 20 µg kg^−1^ (the Swiss MRL) for the samples of Abha and Al Bahah which ranged between 64.74 and 151.06 (µg kg^−1^), respectively. CTC was only detected in two samples from Riyadh with elevated values above the Swiss MRL of 30.99 and 33.60 µg kg^−1^. Notably, SDM and OTC were not found in any of the examined samples (Table 5).

**Table 5:** Concentrations of antibiotics in Saudi Arabian honey

Region	Total/positive samples (frequency %)	Conc	Concentrations (µg kg^−1^)					
			SMT	SMX	SDM	OTC	TC	CTC
Al Makhwah	**3/3** (100)	Average	**2.213**	**6.780**	** < d.l.** *	** < d.l**	** < d.l**	** < d.l**
		Max	3.022	8.102	–	–	–	–
Ha’il	**3/3** (100)	Average	**1.593**	**3.171**	** < d.l**	** < d.l**	** < d.l**	** < d.l**
		Max	3.053	3.366	–	–	–	–
Al Bahah	**3/3** (100)	Average	**36.940**	**4.536**	** < d.l**	** < d.l**	**124.094**	** < d.l**
		Max	38.689	5.666	–	–	151.066	–
Jazan	**3/0** (0)	Average	** < d.l**	** < d.l**	** < d.l**	** < d.l**	** < d.l**	** < d.l**
		Max	–	–	–	–	–	–
Abha	**3/3** (100)	Average	**0.183**	**7.100**	** < d.l**	** < d.l**	**76.533**	** < d.l**
		Max	1.883	7.867	–	–	88.320	–
Riyadh	**3/2** (66.6)	Average	**1.749**	** < d.l**	** < d.l**	** < d.l**	** < d.l**	**32.300**
		Max	3.463	–	–	–	–	33.600
Total	**18/14** (77.7)	Main average	**8.536**	**5.397**	** < d.l**	** < d.l**	**100.313**	**32.300**
Swiss MRL		(µg kg^−1^)	**50**	**50**	**50**	**20**	**20**	**20**

* < d.l.: below the detection limit

Briefly, the antibiotics detected in Saudi Arabian honey can be arranged on the basis of frequency of detection as SMT > SMX > TC > CTC, while the order was TC > SMT > CTC > SMX based on their detected levels.

### Human risk assessment of exposure to SAs and TCs in honey

Data of Table 6 showed that values of the source related hazard quotient (HQ_S_) for each antibiotic detected in honey were markedly lower than the critical value of 1 for all samples from the three countries. This indicated that the aggregated exposure level was lower than the ADI; consequently there were no significant risks associated with the consumption of contaminated honey samples with the detected levels of SAs and TCs at the studied sites. The highest values of HQ_S_ were recorded for TC and CTC in Libyan samples (0.145 and 0.166, respectively) followed by Saudi Arabian samples (0.127 and 0.041, respectively) and then Egyptian samples (0.087 and 0.036, respectively).

**Table 6:** Risk characterization parameters for the detected SAs and TCs in honey samples of Egypt, Libya and Saudi Arabia

Antibiotics	Mean detected conc. (µg/kg)			EDI** (µg)			EXP aggregated (µg)			ADI (µg) ***	HQ_S_			HI_S_			HI_A_		
	Egypt	Libya	Saudi Arabia	Egypt	Libya	Saudi Arabia	Egypt	Libya	Saudi Arabia		Egypt	Libya	Saudi Arabia	Egypt	Libya	Saudi Arabia	Egypt	Libya	Saudi Arabia
SMT	5.19	0.067	8.54	0.014	0.001	0.105	3.76	0.05	8.86	3500	0.001	0.000	0.003	0.006	0.000	0.004	0	0	0
SMX	24.68	< d.l.*	5.40	0.067	–	0.066	17.90	–	5.60	3500	0.005	–	0.002						
SDM	< d.l	< d.l	< d.l	0.000	–	–	–	–	–	3500	0.000	–	–						
OTC	34.81	26.12	< d.l	0.094	0.444	–	66.58	57.82	–	2100	0.031	0.028	–	0.156	0.338	0.168	0	0.003	0.001
TC	96.83	137.21	100.31	0.261	2.333	1.234	185.19	303.71	266.20	2100	0.087	0.145	0.127						
CTC	40.15	157.32	32.30	0.108	2.674	0.397	76.79	348.22	85.72	2100	0.036	0.166	0.041						

* < d.l.: below the detection limit **EDI: Estimated daily intake ***ADI acceptable daily intake for a 70 kg adult per day (FAO/WHO, 2010)

Concerning the source-related hazard index (HI_S_) for mixtures of antibiotics (3 SAs and 3 TCs), it was noted that SAs had higher HI_S_ values than TCs. The highest HI_S_ values of SAs were scored for Libyan samples, followed by Saudi Arabian samples and then those from Egypt, being 0.338, 0.168, and 0.156, respectively. However, notably, HI_S_ values for the SA and TC groups were markedly lower than 1, indicating the safety of the studied samples. Similarly, the calculated values of the adversity-specific hazard index (HI_A_) for the SA and TC groups were also located within safe ranges. These findings revealed that there were no potential adverse effects from the synergistic action of the antibiotics.

The variation in the values of risk characterization parameters among the studied countries was due to the variable detected concentrations plus the level of consumption of honey. For example, Libyan and Saudi Arabian populations have high honey consumption (using it as a sweetener), when compared with the Egyptian population. As well, it should be taking into account that the maximum detected concentrations of antibiotics, such as in samples from Giza and Dakahlia (Egypt), Khoms, Tarhounah, and Masallata (Libya), and Al Bahah (Saudi Arabia), could be ingested through honey.

## Summary and conclusion

In the present investigation, quality assurance of the extraction and screening methods for both HPLC–DAD and HPLC–MS/MS against SAs and TCs revealed high accuracy and selectivity, and good sensitivity. Regarding the survey on the occurrence of SAs and TCs, the results confirmed that SAs antibiotics were dominant in the Egyptian and Saudi Arabian samples, while TCs were the most prevalent in Libya. Although a number of samples had elevated concentrations of SAs and TCs, over the Swiss MRL, the risk assessment study revealed no potential risk of the detected concentrations to consumers’ health. Meanwhile, it should be noted that chronic exposure to antibiotics could represent a potential risk to public health, particularly for those samples with the highest concentrations. Further efforts are needed to limit human exposure to antibiotics through the control of antibiotic application, inspection programs, and encouraging the use of natural alternatives to antibiotics in the production of foods of animal origin. In addition, authorities such as the European Union and US FDA have to set MRLs of these drugs in bees’ honey.

## Supplementary Information

Below is the link to the electronic supplementary material.

Supplementary file1 (DOCX 839 KB)
