# Supplementary material for: Method validation and risk assessment for sulfonamides and tetracyclines in bees’ honey from Egypt, Libya and Saudi Arabia
Source: Environ Geochem Health. 2022 Apr 13;45(3):997–1011. doi: 10.1007/s10653-022-01258-0 (PMC10014665; doi:10.1007/s10653-022-01258-0)
Supplement: Supplementary file 1 — Supplementary file1 (DOCX 839 KB) [file 10653_2022_1258_MOESM1_ESM.docx]

**Supplementary data**

**Method validation and risk assessment for sulfonamides and tetracyclines in bees' honey from Egypt, Libya and Saudi Arabia.**

Ahmed, Mohamed Bedair M. ^1*^, Taha, Amro Ahmed ^2,4^ & Mehaya, Fathy Mohamed Saber ^3^

*^1^Department of Food Toxicology and Contaminants, National Research Centre, Cairo, Egypt.*

*^2^Department of Beekeeping, Plant Protection Research Institute, Agricultural Research Center, Cairo, Egypt.*

*^3^Department of Food Technology, National Research Centre, Cairo, Egypt.*

*^4^ Research and Training Station, King Faisal University, Al-Ahsa, Saudi Arabia.*

**Table S1. Honey samples from different regions of Egypt, Libya and Saudi Arabia.**

| **Country** | **Honey types** | **No. of samples** | **Governorate** | **Honeybee Race** |
| --- | --- | --- | --- | --- |
| **Egypt** | Clover | 17 | Dakahlia, Giza, Kafr El-Sheikh, Suiz,  Sharqia, Qalyubia | *Apis mellifera carnica* |
|  | Citrus | 16 | Qalyubia, Monufia, Ismailia, Beheira | *Apis mellifera carnica* |
| **Libya** | Citrus+multi-flora | 8 | Tripoli | *Apis mellifera saharensis* |
|  | Sider | 7 | Bani Walid &Aziziya | *Apis mellifera saharensis* |
|  | Thyme | 10 | Tarhounah, Gheryan, Khoms, Msallata | *Apis mellifera saharensis* |
|  | Peganum, Harmal | 8 | Commercial | *Apis mellifera saharensis* |
| **Saudi Arabia** | Acacia, Talh | 9 | Hail, Haiya, Taif, Gao | *Apis mellifera jemenitica* |
|  | Acacia,Summra | 5 | Gazan | *Apis mellifera carnica* |
|  | Sider | 4 | Rafhaa | *Apis mellifera jemenitica* |

**Table S2. Gradient of mobile phases used for separation of SAs and TCs by HPLC-DAD and HPLC-MS/MS**

| HPLC-DAD | | | HPLC-MS/MS | | |
| --- | --- | --- | --- | --- | --- |
| Time (min) | A_1_(%) | B_1_(%) | Time (min) | A_2_(%) | B_2_(%) |
| 0 | 10 | 90 | 0 | 96 | 4 |
| 5 | 18 | 82 | 15 | 88 | 12 |
| 10 | 23 | 77 | 25 | 80 | 20 |
| 15 | 25 | 75 | 30 | 96 | 4 |
| 20 | 20 | 80 | 35 | 96 | 4 |
| 25 | 13 | 87 | - | - | - |
| 28 | 10 | 90 | - | - | - |

A_1_: ACN (HPLC grade); B_1_: 0.03 M oxalic acid, pH 2.3

A_2_: 0.1 % formic acid in nanopure water, pH 2.6

B_2_: 0.1 % formic acid in ACN (HPLC grade)

| **Egypt:**  3 = Beheira  5 = Dakahlia  9 = Gharbia  10 = Monufia  11 = Qalyubia  13 = Ismailia  14 = Giza | 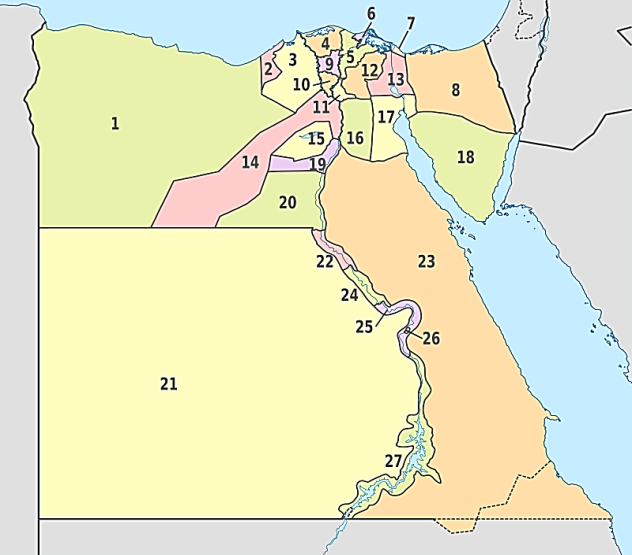 |
| --- | --- |
|  | |
| **Libya:**  2 =  Al Aziziyah  7 = Msallata  7 = Khoms  11 = Wadi Al Hayaa  16 =  Gheryan  20 = Bani Walid  22 = Tripoli  23 = Tarhounah | 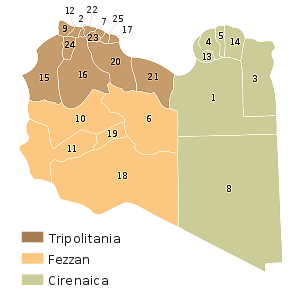 |
|  | |
| **Saudi Arabia:**  3 =  Ha'il  9 = Riyadh  17 = Abha  19 =  Al Bahah  19 =  Al Makhwah  20 = Jazan | 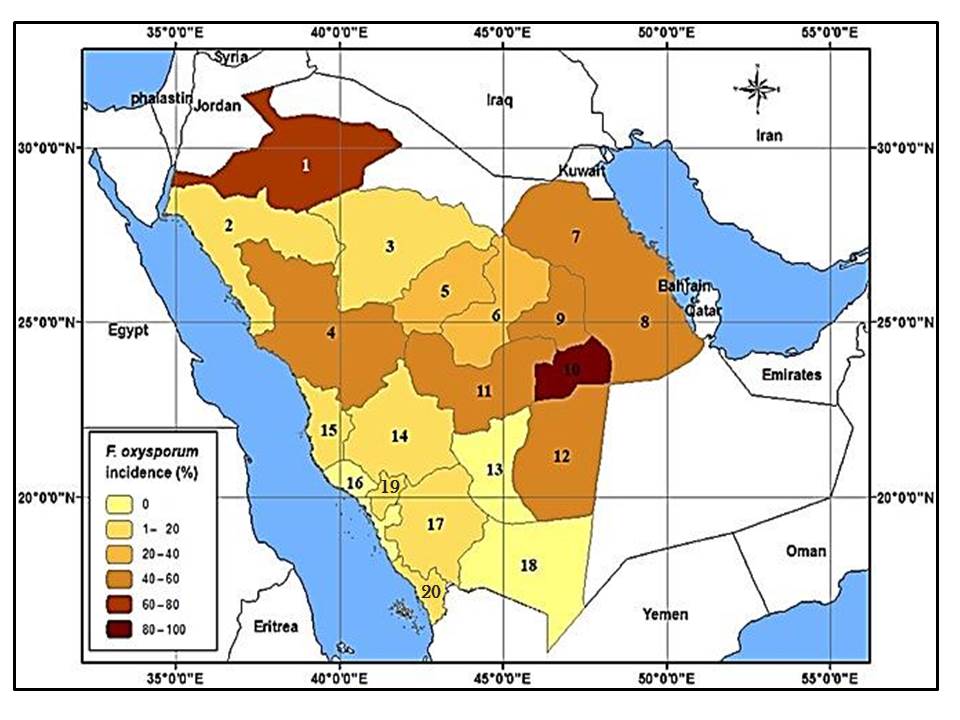 |

**Figure S1. Geographical distribution and key numbers of the selected sites for sampling in Egypt, Libya and Saudi Arabia**


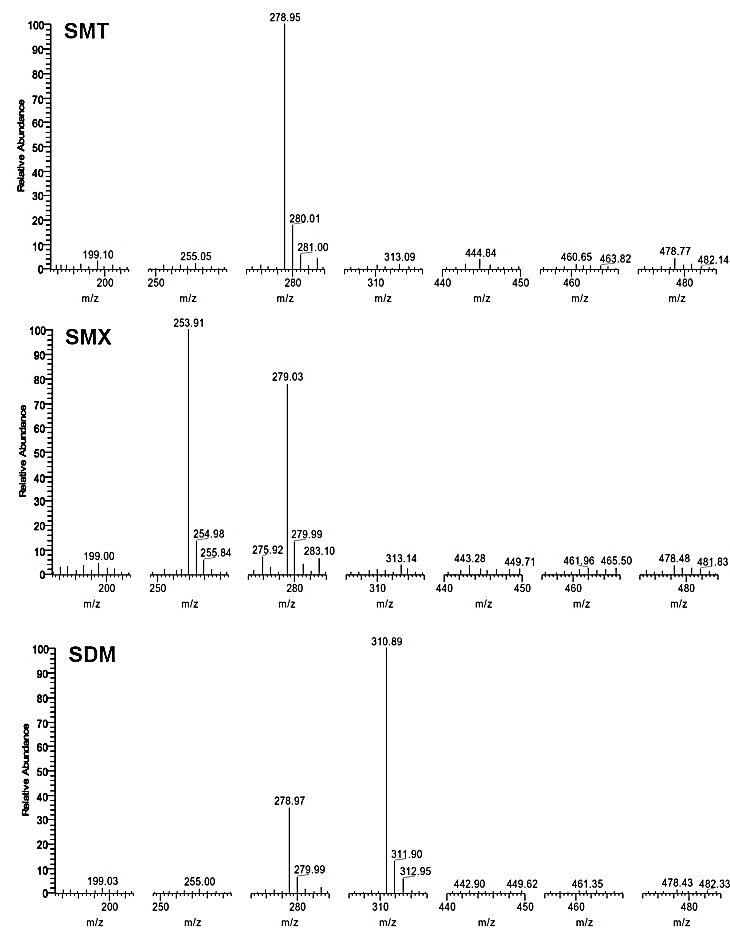


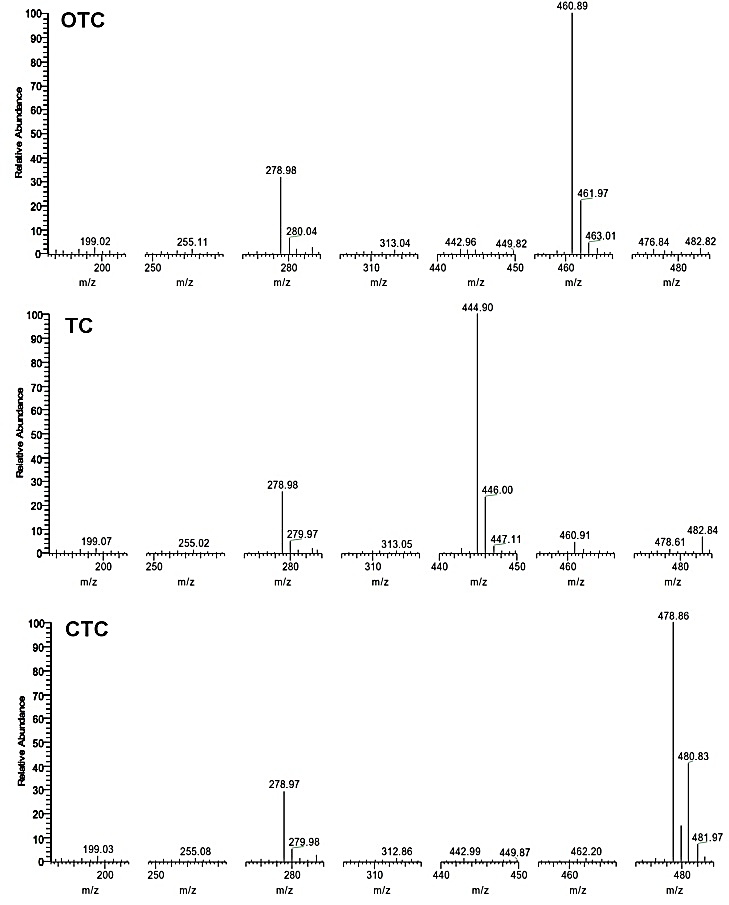


**Figure S2. Fragmentation (Parent compounds of SAs and TCs)**
